# ‘I believe respect means providing necessary treatment on time’ - a qualitative study of health care providers’ perspectives on disrespect and abuse during childbirth in Southwest Ethiopia

**DOI:** 10.1186/s12884-023-05567-9

**Published:** 2023-04-17

**Authors:** Hirut Megersa Werdofa, Lisbeth Thoresen, Belayneh Lulseged, Anne Karin Lindahl

**Affiliations:** 1 School of Nursing, St. Paul’s Hospital, Millennium Medical College, Addis Ababa, Ethiopia; 2 Department of Interdisciplinary Health Sciences, Institute of Health and Society, Faculty of Medicine, University of Oslo, Oslo, Norway; 3 School of Public Health, St. Paul’s Hospital, Millennium Medical College, Addis Ababa, Ethiopia; 4 Department of Health Management and Health Economics, Institute of Health and Society, Faculty of Medicine, University of Oslo, Nordbyhagen, Norway; 5 Akershus University Hospital, Nordbyhagen, Norway

**Keywords:** Provider perspective, Disrespect and abuse, Childbirth, Teaching hospital, Mistreatment

## Abstract

**Background:**

The majority of maternal deaths occur in low-income countries, and facility-based childbirth is recognised as a strategy to reduce maternal mortality. However, experiences of disrespect and abuse during childbirth are reported as deterrents to women’s utilisation of health care facilities. Health care providers play a critical role in women’s experiences during childbirth; yet, there is limited research on service providers’ views of disrespect and abuse in Ethiopia. Therefore, this study aimed to explore providers’ perspectives on disrespect and abuse during childbirth in a teaching hospital in Southwest Ethiopia.

**Method:**

Qualitative study was conducted in a tertiary teaching hospital in Jimma Ethiopia. In-depth interviews were conducted with 32 purposefully selected health care providers, including midwives, obstetrics and genecology resident’s, senior obstetricians and nurses. Interviews were audio-recorded, transcribed and thematically analysed using the qualitative data analysis software program MAXQDA.

**Results:**

Three major themes were identified from the health care providers’ perspectives: (1) respectful and abuse-free care, (2) recognised disrespect and abuse; and (3) drivers of women’s feelings of disrespect and abuse. The first theme indicates that most of the participants perceived that women were treated with respect and had not experienced abuse during childbirth. The second theme showed that a minority of the participants recognised that women experienced disrespect and abuse during childbirth. The third theme covered situations in which providers thought that drivers for women felt disrespected.

**Conclusion:**

Most providers perceived women’s experiences as respectful, and they normalized, and rationalized disrespect and abuse. The effect of teaching environment, the scarcity of resources has been reported as a driver for disrespect and abuse. To ensure respectful maternity care, a collaborative effort of administrators, teaching institutions, professional associations and researchers is needed. Such collaboration is essential to create a respectful teaching environment, ensure availability of resources, sustained in-service training for providers, and establishing an accountability mechanism for respectful maternity care.

## Background

The majority (99%) of maternal deaths worldwide occur in low-income countries, and Sub-Saharan Africa accounts for 66% of maternal deaths [1]. In 2017, Ethiopia documented 401 maternal deaths per 100,000 live births [2]. Facility-based childbirth is recommended as a strategy for reducing the high maternal mortality in the past two decade [3]. In line with this recommendation, the Ethiopian government aims to improve women’s access to and utilisation of facilities for childbirth, and progress has been made towards this goal. For instance, the number of health care facilities and health professionals in Ethiopia has increased in the past decade [4, 5]. In addition, to reduce economic barriers to women’s utilisation of health care facilities for childbirth in Ethiopia, women do not pay for maternal health services provided in public health care facilities [6]. However, despite these efforts, more than half (52% ) of Ethiopian women gave birth at home in the hand of untrained individuals: their relatives or traditional birth attendants [7].

Previous studies have shown that women’s utilisation of maternal health services for childbirth depends on their perceptions of the quality of interpersonal care during childbirth [8, 9]. Given that providers have a crucial role in lifesaving clinical care and the quality of interpersonal care during childbirth [10, 11], women’s perceptions of maternal health services and their inclination to suggest using such facilities to other women partly depend on their trust in providers [8].

Disrespect and abuse in health care facilities are known as barriers to women’s utilization of facilities for childbirth [12]. This include women’s encounters or facility settings that are perceived to be undignified and that local consensus finds to be humiliating [13]. Bowser and Hill’s landscape analysis classified disrespect and abuse during childbirth into seven types: physical abuse, non-dignified care, neglected care, verbal abuse, non-confidential care, detention in a health care facility and discrimination [14].

Disrespect and abuse during childbirth is reported as a violation of a woman’s right to good quality and dignified intrapartum care [15]. In Ethiopia a recent study of providers reported that disrespect and abuse as (1) a professional standards violation, (2) a way providers demonstrate power over women in health care facilities to overcome their feeling of inferiority related to urban, educated, and rich women and (3) a weakness of health care facilities [16]. The degree of disrespect and abuse was found to vary with the study setting and to be more common in hospitals than in health centres in Ethiopia. For example, a study in Addis Ababa, Ethiopia, shows that neglected care was reported by 14.1% of women in health centres and 63.6% in hospitals [17]. Additionally, a study in the western part of Ethiopia indicate that women who gave birth in a health centers reported disrespect and abuse less frequently than women who give birth in hospital [18]. Another study in Ethiopian public health facilities found that health centres provided more respectful maternity care than hospitals [19].

Women’s experiences of disrespect and abuse during childbirth in health facilities have received global attention in the past ten years [15, 20]. The White Ribbon Alliance (WRA) developed a respectful maternity care charter [20], and the World Health Organization (WHO) endorsed a statement to combat disrespect and abuse during childbirth [15]. In 2015, Ethiopia developed strategies to on a caring, respectful and compassionate (CRC) health workforce via a five-year health system transformation plan [5]. However, women still experience disrespect and abuse during childbirth in Ethiopia [9] and in many other countries [21, 22] even though every woman deserves and has the right to receive quality care during childbirth in health care facilities [23]. Prior studies in sub-Saharan Africa show the severity of the problem. The magnitude of disrespect and abuse in Sub-Saharan Africa ranges from 20% in Kenya [24] to 98% in Nigeria [25]. Similarly in Ethiopia the magnitude varies from 36.6% [19] to 98%[26] recent pooled prevalence of 49%[27]. A study in Ghana reported that 72% of midwifery student reported disrespect and abused practice [28]. Qualitative studies on the provider perspective in sub-Saharan Africa show the drivers and extent of disrespect and abuse [29, 30]. Midwifery students in Ghana, for example, reported rationalization of disrespectful and abusive care; the culture of blame for poor maternal outcomes as a driver of disrespect and abuse [29]. A Tanzanian study looked at midwives’ practices and found both respectful and disrespectful care [30]. According to a study conducted in Zambia, on provider perspective on behavioral barriers for respectful practice reported were believing what they are expected to do, normalizing disrespect and abuse, believing that the costs of providing respectful care outweigh the benefits, and believing that they do not need to provide respectful care [31].

The provision of quality maternal health services demands a well-functioning health care facility and qualified health care providers who can deliver effective, safe and compassionate care [32, 33]. It is reasonable to conclude that the competence and attitudes of health care providers and the type of health care setting play critical roles in the quality of care and women’s experiences during childbirth. In Ethiopia, most studies have focused on women’s perspectives [9, 34, 35], with few qualitative studies conducted on providers and their views, experiences and attitudes. Among the existing qualitative studies of providers’ perspectives on disrespect and abuse in Ethiopia, none have investigated disrespect and abuse in the context of a tertiary teaching hospital where providers from various professions and students work in the labour ward [16, 36]. Therefore, this study aimed to explore health professionals’ perspectives on disrespect and abuse in a tertiary teaching hospital in Southwest Ethiopia.

## Method

### Study design and period

A qualitative study was conducted from January to February 2018 to obtain health care providers’ perspectives on women’s experiences of disrespect and abuse during childbirth.

### Study setting

The study was conducted in Jimma University Medical Centre a tertiary teaching and referral hospital located in Jimma city 352 km southwest of Addis Ababa, Ethiopia. The Jimma University Medical Centre serves approximately 20 million people who reside in its catchment area. The population is diverse and consists of people from the three regional states of: Oromia, Southern Nations Nationalities and Peoples (SNNP) and Gambelia, as well as from a nearby country: South Sudan. The obstetrics department had eight consultant obstetricians, 33 resident physicians from year one to year four and 75 staff midwives and nurses during the data collection period. There were also final-year undergraduate medicine students (medical interns) and midwifery and nursing students working in the department during the data collection period.

### Study population

The study population included: Physicians (residents and senior obstetrician), midwives, nurses working in Jimma university medical centre. We interviewed who had at least six months of work experience in the labour ward and were working in the obstetric unit (i.e. labour ward, maternity ward, maternity operating theatre and maternity recovery room) of Jimma University Medical Center (Table 1). We sampled participants purposively based on the above inclusion criteria. The first author (HMW) and a research assistance identified potential participants who met the inclusion criteria while conducting participant observation, explained the purpose of the study to the potential participants and obtained their written consent prior to their participation in an interview. The interviews ended after 32 participants had been interviewed, as there were no new information emerging.

**Table 1:** The sociodemographic and service-related characteristics of the participants who were working in a teaching hospital in Southwest Ethiopia

Characteristic	Category	N = 32
**Sex**	Male	17
	Female	15
**Age (years)**	22–24	7
	25–29	18
	30 and above	7
**Service period (years)**	1–2	6
	3–5	17
	6–9	4
	10 and above	5
**Profession**	Midwife	20
	Physician (residents and obstetrician)	7
	Nurse	5

### Data collection

Data were collected through face-to-face in-depth interviews using a semi-structured interview guide. Semi-structured in-depth interviews are commonly used in qualitative research and it allows to gather open data, examine participants’ thoughts, feelings, and beliefs about specific topics, and explore deeper and sometimes sensitive issues [37] .

The guiding questions were developed based on the study’s objectives and existing literature on disrespect and abuse. The guide included probing questions that ensured an exploration of the participants’ views on women’s possible experiences of disrespect and abuse during childbirth. The interview guide was pre-tested with two health care providers (a midwife and a resident) to ensure the appropriateness of the guiding questions. The first author, (HMW), who has clinical experience in maternity and reproductive health and academic experience in midwifery and nursing education, conducted the interviews. The interviewer does not work at the study hospital; hence, she had no direct influence on the participants’ responses or the study setting. Each interview was conducted in the participant’s local language, Amharic. The interview duration was between 40 and 45 min, and each interview was audio-recorded with the participant’s consent. The interview venue (the study hospital), date and time were all scheduled to suit the participants.

### Data analysis

Two authors, HMW and BL (co-author with experience in qualitative research and with qualitative software training), both local language natives, conducted the analysis. First, HMW transcribed the data verbatim, and then BL checked the transcripts. Two translators (who have English language expertise) translated the transcripts from the local language into English, and HMW and B.L verified them for consistency. Braun and Clarke’s thematic analysis method was utilised, which consists of a six-step iterative process of (1) familiarisation, (2) coding, (3) generation of initial themes, (4) reviewing themes, (5) defining and naming themes and (6) writing-up [38, 39]. Thematic analysis allow researchers to immerse themselves in the data and generate themes inductively from the data’s. Furthermore, it allows to gain rich and useful insights into the context. It is useful for developing more nuanced interpretations of a phenomenon under study [38, 39].

HMW and BL repeatedly read the data to become familiar with it, understand it and become fully immersed in it. For the coding step, HMW and BL independently coded the data and then discussed their findings until they reached an agreement. Initial themes were produced inductively from the data. When developing the initial themes, the two authors HMW and B L iteratively assessed all the codes to identify patterns and then grouped (categorized) those patterns into more comprehensive patterns of meaning into themes that had been defined. Then, in the review step, the identified themes were checked and refined for accurate representation, overlap and broadness. The final themes were described during the defining and naming themes step. The final themes were interpreted according to the Bowser and Hill disrespect and abuse categories [14], and a codebook was developed. Thus, the analytical process was six step thematic analysis method. The MAXQDA qualitative analysis software program was used to organise data [41]. The two co-authors AKL (Physician researcher and expert of quality and patient safety) and LT (nurse and qualitative researcher) reviewed, discussed and approved each stapes of data analysis.

In this study trustworthiness of the findings assured base on : confirmability, transferability, dependability and authenticity [41–43] : Purposive sampling techniques were employed; participants from three different professions and with different experiences in the maternal health service were enrolled in the study to ensure authenticity. To ensure conformability of our finding we conducted member checking on the interview transcript by involving three participants from the three disciplines: midwifery, physician (resident), and nursing, before final conclusion. In addition, the data was independently analysed, and each evolving theme was discussed, and the differences resolved at each step of data analysis to ensure dependability. Description of the study method: Setting, participant selection, data collection, and data analysis ensured transferability.

The Consolidated Criteria for Reporting Qualitative Research (COREQ) checklist was used to guide the reporting of this study [44].

Anonymity of data was ensured by using a number for each transcript file instead of a name and other identifying information, and the transcripts were kept in a password-protected folder on the researcher’s (HMW) personal computer.

### Ethical consideration

#### Ethics approval and consent

The Regional Committee for Medical and Health Research Ethics of South East Norway (REC), section B (ref 2017/1050b) and the Institutional Review Board of St. Paul’s Hospital Millennium Medical College, Addis Ababa, Ethiopia (reference number PM23179/25/9/2017) provided ethical approval for this study. The two ethical approval letters (from REC and St. Paul’s Hospital Millennium Medical College Institutional Review Board) and a support letter were submitted to the study hospital and permission was obtained from hospital Medical director. The study was voluntary and participants who agreed to take part in the study signed informed consent form. The audio interviews data and transcripts were numbered to protect the participants’ confidentiality and participants were given the assurance that their anonymity would always be respected and protected. All methods were performed in accordance with the relevant guidelines and regulations.

## Results

### Participants’ sociodemographic and service-related characteristics

Thirty-two health care providers were included in the study until data saturation (Table 1). Of the 32 participants, five were working as both clinical and administrative staff.

### Providers’ perspectives on women’s experiences of disrespect and abuse during childbirth

The analysis of the interviews with the providers yielded three main themes. First, most of the participants perceived that women were treated with respect and did not experience abuse during childbirth. However, the second generated theme showed that a minority of the participants recognised that women experienced disrespect and abuse. The third theme drivers for women feeling of disrespected (Table 2). Most of the participants believed that women experience their care during childbirth as respectful and free of abuse. More midwifes than other health care workers perceived that women experience care as respectful and free of abuse, while more residents than other health care workers perceived that women experience disrespect and abuse during childbirth in some instances. Most female participants reported that women are not disrespected during childbirth, whereas most male participants reported that women sometimes experience disrespect and abuse and feel disrespected (Table 2).

**Table 2:** Themes and sub-themes of the providers’ perspectives on women’s experiences of disrespect and abuse during childbirth in a tertiary teaching hospital

Main theme	Sub-theme
Respectful and abuse-free care	Timely clinical care
	Abusive- free care
Recognised disrespect and abuse	Non-consented procedures
	Insufficient information
	Procedures performed without privacy
	Verbal abuse of women
	Neglect
Drivers of women’s feeling of disrespect and abuse	Teaching environment
	Health system constraints
	Unfulfilled expectations and preference

#### Respectful and abuse-free care

Most of the participants perceived that women were neither disrespected nor abused during childbirth; instead, they perceived that the women received respectful and abuse-free care. Some participants connected respectful care to timely care.

##### ***Timely care***

Some participants perceived respectful care as timely clinical care and the fulfilment of a woman’s physiological needs during labour and birth. These participants believed that women were not disrespected and abused when giving birth in the study hospital, and cited prompt care for their perception. They reported their own practice and that of their colleagues as timely, routine clinical care that women received during labour. For example:

*‘I think our service is respectful. In this unit, women are not disrespected and abused during labor. We strive to provide timely care while women are admitted here for childbirth. You see, we check their health status as soon as they come to this labor ward, and we provide the necessary care right away.’(Participant code 7, Midwife female).*

Some participants viewed respectful care as timely care for obstetric complications. They reported routine provision of necessary services based on a woman’s need at admission; thus, they believed disrespect and abuse of women during childbirth was unlikely in the study hospital, as the following quote describes:

*‘Usually, when they (women) arrive at this labor ward with normal labor or obstructed labor, we give all essential care. Therefore, I believe respect means providing necessary treatment on time.’(Participant 17, Resident, Male)*

#### Abuse-free care

Some participants perceived women’s experiences as abusive-free because they viewed the absence of verbal and physical abuse as the only indicator of respectful care. On the other hand, some participants witnessed and perpetrated verbal abuse and reported justifications for their practice and believe women experience as respectful.

A minority of participants believed that women did not experience disrespect and abuse during childbirth because they did not perpetrate or observe verbal and physical abuse of women during labour. These participants evaluated their practice and women’s experiences during labor and childbirth based on the presence or absence of verbal and physical abuse; they did not consider other forms of disrespect and abuse in relation to women’s experiences and their practice. For example, one participant said:

*‘We don’t hit or yell at women during labor, so I do not think women experience disrespect and abuse during labor in this hospital. Instead, I think women have respectful experience during labor.’ Participant 8, Midwife, Female,)*

”*Yah I think our care for women during labor, respectful, because no shouting, scolding hitting women during labor in this unit " ( Participant 25 ,Midwife ,female )*

On the other hand some participants justified their own practice of yelling at women by highlighting how some women refused to listen to the health care professionals during labour. Those participants perceived yelling at a woman as a consequence of the woman’s behaviour and the providers’ intentions for the safety of the woman and her baby. Due to these beliefs, they did not regard this practice as disrespect and abuse, with one participant saying:

*‘In the labor ward, women are not disrespected and abused during labor. I can say that our care for the women was respectful. We yell at an uncooperative woman during labor due to her uncooperative behavior and to save the life of her unborn baby.’ (Participant 6, Midwife, female)*

Some participants explained that the use of inappropriate words by providers during labour should not be perceived as disrespect and abuse. Instead, they perceived it as the effect of workload-related stress on the provider, as the following quote demonstrates:

*‘I don’t think women experience disrespect and abuse during childbirth in our unit. I believe our care is respectful. However, the work burden sometimes disappoints us, and we yell at women when they are uncooperative for our instructions.’ (Participant1, Midwife, Male )*

#### Recognised disrespect and abuse

Participant residents and a minority of the participant midwives believed that women sometimes experience disrespect and abuse during childbirth. They identified their own and colleagues’ practices of performing non-consented procedures, insufficient information for women before procedures, procedures performed without privacy, verbal abuse of women and neglect as examples of disrespect and abuse.

##### ***Non-consented procedures***

In terms of performing non-consented procedures, one participant shared the following insight:

*‘We’ perform some of the procedures during labor respectfully to women, and the other may be disrespectful. In particular, in the second stage of labor, women cry due to labor pain; and procedures are performed without consent.’(Participant 23, Midwife, Male)*

##### ***Insufficient information***

Some participants perceived that women may receive partially disrespectful care; for instance, there may be a lack of sufficient information provided to them before treatment. They reported how providers performed caesarean sections on women without providing adequate information about the risks of the procedures. One participant said:

*‘To some extent, our care is respectful, but I cannot say it is completely respectful care because some of our practices are disrespectful. For example, a cesarean section is performed without sufficient explanation of the procedure’s risk to the woman and her family.’ (Participant 32, senior obstetrician, Male)*

##### ***Procedures performed without privacy***

Some participants believe women experience disrespect and abuse during childbirth due to violation of privacy while they admitted in the labour ward. They had witnessed physical examinations being conducted in view of numerous students while the women were in the first stage of labour. Participants emphasised the scarcity of resources and the presence of students as contributing factors to this lack of privacy, with one participant saying:

*‘Women’s privacy has not been maintained, so I cannot say our service is completely respectful. For instance, vaginal examination is performed in view of many students and other providers in the first stage room due to the scarcity of curtains.’ (Participant 26.Midwife, Male )*

##### ***Verbal abuse of women***

A minority of participants reported that women sometimes experienced verbal abuse during childbirth and they perceive such women experience as disrespectful. For instance, they reported the practice of provider’s yelling when a woman could not understand their concerns and when a woman’s actions disappointed them. In addition, providers yelled at women when they were attempting to ensure the safety of the childbirth process. These participants perceived the verbal abuse that women sometimes faced during labour and childbirth as disrespectful, as the following quote demonstrates:

*‘Women are occasionally disrespected during labor and childbirth because we sometimes yell at women when we perceive that they cannot understand our concern for a safe delivery.’ (Participant 29, Resident, Male)*

##### ***Neglect***

Some participants reported that there were improvements in the care of women in the labour ward and perceived the provided care as both respectful and disrespectful. In these circumstances, participants linked women’s poor experiences with the negligent behaviour of providers. For example, a nurse reported that women’s experiences of disrespect were in part due to the negligence of some providers:

*‘It is partially disrespectful. When we compare current practice of providers with the previous practice of providers, it is good. However, my coworkers do not have the same response toward their responsibility; some of them are negligent. Sometimes, due to the negligence of some providers, fatal consequences happen to women and their babies’ (Participant 18, Nurse, female)*

### Drivers for women feeling of disrespect and abuse

Participants highlighted circumstances that occurred during labour and birth that made women feel disrespected. More than half of the participant physicians (residents and obstetricians) and a minority of the participant midwives reported that women’s feelings could be related to the teaching environment, health system constraints, *failure to full fill women expectation and preference*.

#### Teaching environment

According to one participant, receiving care in a teaching hospital had significant consequences on women experience:

*‘Sometimes women feel disrespected because this hospital is a teaching hospital, and the environment may not be comfortable for them.’ (Participant 11, Midwife Male).*

For example, participants perceived that women’s feelings of disrespect could be related to undergoing frequent examinations conducted by students. Those participants perceived this practice as a deviation from the recommended standard procedure in addition to a driver for women’s feelings of disrespect. One participant shared:

*‘Yeah, women feel disrespected due to the teaching environment that leads to too frequent vaginal examinations that is performed less than 4 hours apart. However, vaginal examinations are recommended every four hours during labor.’(Participant 20, Midwife, Male)*

#### Health system constraints

Some participant reported scarcity of resource make women to feel disrespected. One of the participants reported women’s feelings of disrespect being due to a lack of a bed at admission. As the participant reported, when there is a large number of admissions, there is a bed shortage:

*‘I think there are some circumstances that make women feel disrespected because the hospital has a high caseload and have scarcity of resources. Sometimes women could not get a bed immediately upon admission. This experience may make women feel disrespected.’ Participant 21, Nurse, Male).*

#### Unfulfilled expectations and preference

Some participants consider maintaining privacy as one of women’s expectations, and they consider not fulfilling women’s expectations as a driver of women’s feelings of disrespect. The participants described how women could feel disrespected due to their unfulfilled expectations of privacy during their admission to the hospital, as the following quote shows:

*‘I think the women felt their experience in the hospital was disrespectful because they expect physical privacy during labor. However, when they were admitted to labor wards, we could not protect their privacy while we are doing procedures.’ Participant 20, Midwife, Male).*

Participants also said that not respecting women’s preferences made them feel disrespected. For example, they mentioned that it was disrespectful to not respect a woman’s preference for the mode of delivery, with one participant saying:

*‘Women may feel disrespected because sometimes their choice is not respected. For example, in this unit when a woman wants a cesarean section, her choice was not accepted unless she has an indication.’ Participant 24, Midwife, female)*

## Discussion

The aim of this study was to explore the perspectives of health care providers on women’s experiences of disrespect and abuse during childbirth in a tertiary teaching hospital in Southwest Ethiopia. Three major themes emerged from the providers’ perspectives: respectful and abuse-free care, recognised disrespect and abuse and drivers for women feeling of disrespect and abuse. Most of the providers who participated in the present study perceived the care that women received during childbirth as respectful. However, previous quantitative [34] and qualitative [35] studies found that women reported disrespected and abused during childbirth. A study conducted in Kenya reported similar discordance between women’s and providers’ opinions [45].

Some providers in this study believed women do not experience disrespect and abuse during childbirth, Most of those participants reported timely clinical care as a package of respectful care. However, respectful maternity care in addition to right to timely care includes dignity, respect, privacy, autonomy, right to information and informed consent, and being free from harm and ill-treatment [20]. This perception of providers may be linked with concept of safe motherhood that focuses on physical safety [20]. Another explanation may be that in the maternal health service settings, providers have been made accountable for pregnancy outcomes but not for the women’s experiences [46]. In fact, lack of an accountability mechanism for respectful maternity care was reported as a contributor to disrespect and abuse in a prior study [22].

In the present study, providers who recognized disrespect and abuse only reported non-consented care, lack of privacy, verbal abuse, and neglect as forms of disrespect and abuse practices out of the seven known disrespect and abuse practices [14]. However, a prior quantitative study on women in the same facility reported seven forms of disrespect and abuse [34]. In addition, this difference might support the Freedman definitions, in which “normalized experience” is an experience that women consider disrespect and abuse and that may be normalized by their health care providers [13].

The minority of participants in the current study witnessed and perpetrated verbal abuse of women; however, they did not perceive this as a disrespectful act towards the women. Those participants considered verbal abuse during labour as a consequence of a woman’s behaviour, and they emphasised that it was motivated by a provider’s intention to create a safe birthing process. Similar findings have been reported in prior studies in which provider’s normalised verbal abuse of women [47, 48]. A previous qualitative study conducted in Ethiopia found that midwives normalised verbal abuse, physical abuse and non-consented care, and the authors of that study mentioned the provider’s practices as intended for good outcomes [36]. In contrast to those this study finding, a quantitative study that involved women at the study hospital showed that 88.6% of women reported physical abuse as disrespect and abuse [34].The differences between the views of women in labour and the views of providers are of particular interest. Improvements in attitudes and, thus, care may result in more women seeking to give birth in health care facilities, as is the recommended practice [49].

Moreover, participants who perceived women as being disrespected during childbirth reported routine violations of women’s privacy that resulted from the presence of students and the scarcity of curtains. This finding aligns with those of prior qualitative and quantitative studies conducted in Ethiopia [34, 35]. The consistent practice of disrespect and abuse due to the situation of clinical setting and scarcity of resource leads to normalisation of poor practice [36]. In addition, such practices may have implications for the behaviour of the students assigned to the study hospital [14, 36, 50].

Some participants reported that women felt disrespected due to the excessive number of physical examinations they underwent in teaching environment. These findings agree with those of a systematic review that highlighted the objectification of women [12] and those of a prior qualitative study that involved women in Ethiopia [35].

In a teaching hospital, one would expect the health care providers to maintain high standards and have knowledge-based and respectful attitudes and behaviours towards women giving birth. It is also reasonable to expect providers in teaching hospitals to be aware of the responsibility associated with being a role model to medical, nursing and midwifery students.

Even though our study was conducted in a hospital findings contrasts with those of other studies conducted in other regions and facilities in Ethiopia in which disrespect and abuse during childbirth have been reported to occur more often in hospitals than in health centres [17, 18]. The authors of those studies claimed that hospitals have high caseloads and staff shortages, which make it difficult to provide adequate care. This difference in findings might be due to variations in the characteristics of the facility, method and study participants in the different studies. For instance, in Ethiopia, hospitals are categorised as district, general, and specialised or teaching. Non-teaching hospitals in Ethiopia are managed by full-time employee providers and sometimes face staff shortages. However, in teaching hospitals, in addition to full-time employee health care providers, trainees from diverse professions (e.g. residents, medical interns and trainee nurses and midwives) are assigned. Our study was conducted in a teaching hospital in which trainees from diverse professions were assigned and provided service in the same unit, and a shortage of staff may be unlikely.

Most participants in this study who perceived women as not being disrespected were female rather than male providers, which may partly indicate the relationship between gender and disrespect and abuse. Similarly, in another study in Ethiopia, male providers practice more respectful care than female providers [19]. Evidence suggests that female midwives experienced moral distress and burnout as a result of challenges in reproductive, economic, social, and workplace, and that these challenges contributed to their poor professional behaviour [51].

Provider related factors, the teaching environment, a lack of resources main contributing factors to recognized disrespect and abuse practice as contributor for women felling of disrespect and abuse in this study. These aspects may need to be addressed to improve women’s experiences and increase their willingness to give birth at a health care facility.

### Limitations of the study

Most of the participants in the present study were midwives. The findings may therefore be more relevant to midwives than to the other professions. Disrespect and abuse are sensitive topics to discuss with providers. Therefore, providers may have underreported their practice (social desirability bias).

### Strengths of the study

Despite the above limitations, the richness of the findings is a strength of this study. The use of purposive sampling enabled us to capture the perspectives of various professionals. Furthermore, this study is one of the few qualitative studies conducted in Ethiopia that provide findings on the viewpoints of health care providers on disrespect and abuse in a tertiary teaching hospital.

The participants in this study included health care providers from different professional. As a result, findings related to their perceptions of disrespect and abuse may be transferred to various Ethiopian health care settings. Our research team included midwives, a quality, and patient safety expert physician, a qualitative nurse researcher, and a public health professional, which aided in the interpretation of the findings.

## Conclusions and recommendations

In this study, most of the providers (midwives and nurses did not acknowledge women’s experiences of disrespect and abuse during childbirth. This implies that midwives and nurses might have normalized poor practice due to repetition. The providers’ failure to recognize disrespect and abuse practice led them to continue it and failed to consider it as a problem in maternal health service. These might have a great impact on the quality of women’s experiences and shape the behavior of students who are usually assigned to observe and learn clinical skill in teaching hospitals.Therefore, implementation of consistent ongoing in-service and pre-service training on may improve provider believes of disrespect and abuse and enable providers to recognise it when they see it. Accountability mechanism for respectful maternity care at the unit level, might enable the provider to consider it an essential component of maternity service and give attention to it.

Most participants reported that the teaching environment resulted in violations of privacy and result excessive examinations that made women feel disrespected. Excessive examinations in front of numerous students without privacy may imply objectification of women. Stakeholders such as educational institutions, hospital administrator’s professional associations, and interested partners must design strategies to create a non-abusive teaching environment. This might include: alternative teaching approaches such as high-fidelity simulators for sensitive examination; developing guidelines and policies on respectful care that could be specifically implemented in teaching hospitals to improve women’s experiences as well as clinical learning.

The relationship between gender, disrespect, and abuse may be an area of future quantitative research with a large sample size. Furthermore, existing gender differences in perspective and practice of disrespect and abuse necessitate designing interventions at the community, educational institutions and health care facilities to improve respectful maternity care practice.

Interventions that ensure the consistent availability of supplies and equipment may improve women’s experiences related to the scarcity of resource. A large-scale quantitative study of providers’ perspectives is important for a better understanding of providers’ perceptions of disrespect and abuse. Therefore, improving women’s experiences during childbirth and provider practice in health care facilities in Ethiopia demands the efforts of managers, teaching institutions, professional associations and researchers through a holistic approach.

## Data Availability

The datasets collected and/or analysed in the current study are available from the corresponding author on reasonable request.
